# Supplementary material for: Peptosome Coadministration Improves Nanoparticle Delivery to Tumors through NRP1-Mediated Co-Endocytosis
Source: Biomolecules. 2019 May 5;9(5):172. doi: 10.3390/biom9050172 (PMC6572427; doi:10.3390/biom9050172)
Supplement: Supplementary file 1 [file biomolecules-09-00172-s001.pdf]

Peptosome coadministration improves nanoparticle delivery to tumors through NRP1-mediated co-endocytosis

Zhichu Xiang,[1,2,3] Gexuan Jiang,[1,2,3] Xiaoliang Yang,[1,2,3] Di Fan,[1,2,3] Xiaohui Nan, [1,2,3] Dan Li, [1,2,3] Zhiyuan Hu,[1,2,3,4*] Qiaojun Fang[1,2,3,4,5*]

[1]CAS Key Laboratory for Biomedical Effects of Nanomaterials & Nanosafety, National Center for Nanoscience and Technology, Beijing 100190, China;

[2]CAS Center for Excellence in Nanoscience, National Center for Nanoscience and Technology, Beijing 100190, China;

[3]University of Chinese Academy of Sciences, Beijing, 100049, China;

[4]Sino-Danish Center for Education and Research, Beijing, 101408, China;

[5]Beijing Key Laboratory of Ambient Particles Health Effects and Prevention Techniques, National Center for Nanoscience and Technology, Beijing 100190, China

[*]Correspondence authors:

Qiaojun Fang, National Center for Nanoscience and Technology, Beijing 100190, China.

Phone: +86-10-82545562; Fax: +86-10-82545643; E-mail: fangqj@nanoctr.cn.

Zhiyuan Hu, National Center for Nanoscience and Technology, Beijing 100190, China.

Phone: +86-10-82545643; Fax: +86-10-82545643; E-mail: huzy@nanoctr.cn.

KEYWORDS: peptosome, self-assembly, tumor penetration, coadministration, co-endocytosis

Supplementary

Materials

The octadecanoic acid chain (C18) modified amphiphilic peptide PA, peptide iRGD and control peptide PC were synthesized by Top-peptide Bio Co., Ltd. (Shanghai, China) using a Fmoc strategy solid phase peptide synthesis (SPPS) method. Magnetotactic bacteria, *M. gryphiswaldense* (MSR-1), were provided by China Agricultural University. Fluorescein isothiocyanate (FITC), Hoechst 33342, (3-(4, 5-dimethylthiazol-2-yl)-2, 5-diphenyltetrazolium bromide (MTT), Tetramethylrhodamine isothiocyanate mixed isomers (TRITC), Poly(D,L-lactide-co-glycolide) (PLGA, MW 7−17K), Lecithin and dimethyl sulfoxide (DMSO) were purchased from Sigma Aldrich (St. Louis, MO, USA). Phosphate-buffered saline (PBS), Dulbecco's modified eagle medium (DMEM) and RPMI 1640 medium were purchased from Hyclone. Fetal bovine serum (FBS) was purchased from Gibco. Doxorubicin (DOX) was purchased from KeyGEN Biotech (Jiangsu, China). DiR (DilC18(7)) and Cy5 were purchased from Life Technologies (USA). Transtuzumab was purchased from Genentech (USA). All antibodies used in this study were purchased from Cell Signaling Technology (CST, USA). 1,2-distearoyl-sn-glycero-3-phospho -ethanolamine-N-[methoxy(polyethylene glycol)-2000] (DSPE-PEG) was purchased from Avanti (Alabaster, AL). Fe_3O_4 nanoparticles were purchased from Nanoeast Biotech (Nanjing, China)

Methods

Self-assembly of amphiphilic peptide and peptosome construction.

For the construction of APPA and APPC self-assembled peptosomes, 1 mg of peptide and the compounds to be encapsulated (DOX: 0.05 mg, DiR: 0.02mg, TRITC: 0.1mg) were dissolved in 10 μL DMSO. The peptide and the compound solution were mixed together. 1 mL of PBS was added

to the mixture and ultrasonicated (50 W) for 10 min, followed by incubation at room temperature for 1 h. The final solution was centrifuged at 5500 g for 5 min and the aqueous solution was collected. Transmission electron microscopy (TEM) was used to characterize the morphology and the size of APPA and APPC self-assembled peptosomes.[1] Briefly, the collected aqueous solution was dropped onto a carbon grid, dried, and negatively stained with uranyl acetate for TEM. Dynamic light scattering (DLS, Zetasizer Nano ZS90, Malvern) was used to check the size distribution and surface zeta potential of the self-assembled nanoparticles.

Preparation of magnetotactic bacteria and magnetosomes.

The magnetotactic bacteria, MSR-1, were grown in a shaking flask at 30 ℃, 100 r/m with the culture medium as described in our previous study.[2] The bacteria were harvested by centrifugation at 8000 g for 15 min and the supernatant was discarded. To extract magnetosomes, 1 mL of cell pellet was suspended in 2 mL PBS (10 mM, PH 7.4) and sonicated for 26 min at 3 s per time with an interval of 5 s at 200 W to lyze the cells. Afterwards, the suspension of magnetosomes was magnetically separated using a strong magnet, then the supernatant was removed and the separated magnetosomes were resuspended in 3 mL PBS (10 mM, PH 7.4) and sonicated for 13 min at 3 s per time with the time interval of 5 s at 120 W. The suspension was placed on the magnet to separate the magnetosomes. This procedure was repeated twice with the power changing to 80 W and 40 W, respectively. The magnetosomes were washed 3 times with PBS (10 mM, PH 7.4) and characterized with TEM. Dynamic light scattering (DLS, Zetasizer Nano ZS90, Malvern) was used to check the size and surface zeta potential of magnetosome nanoparticles.

Preparation of iRGD-Cy5, LipoDOX and LipoCy5.

The Cy5-NHS was used to label iRGD peptide by the reaction of NHS with the amino group of iRGD in sodium bicarbonate buffer (PH 6.5). The Doxorubicin (DOX) and Cy5 loaded

liposomes were prepared based on a modified single step preparation method.[3] Stock solutions of DSPE-PEG and lecithin were prepared separately at the concentrations of 1 mg/mL in 4% ethanol aqueous solution. Stock solutions of PLGA were prepared at the concentration of 2.5 g/mL in acetone. DOX and Cy5 were dissolved in water at the concentrations of 1 mg/mL and 0.1 mg/mL, respectively. The stock solutions of DSPE-PEG and lecithin were added into ddH_2O and then the PLGA solution mixed with DOX or Cy5 was carefully pipetted into the resulting aqueous solution under sonication for 20 min at 100W. The ratio of the used amount of aqueous solution to that of organic solution was 10:1. The prepared nanoparticles were purified and washed three times with a centrifugal filter (MW: 10K) and characterized with TEM. Dynamic light scattering (DLS, Zetasizer Nano ZS90, Malvern) was used to analyze the size and surface zeta potential of the prepared nanoparticles.

Plasma half-life comparison of peptosome PADiR and peptide iRGD.

To compare the plasma half-life of peptosome PADiR and peptide iRGD, free Cy5, PADiR and iRGD (Cy5 labeled) were intravenously injected into three 5−7 week-old female BALB/c nude mice, respectively. 30 μL of blood from each mouse was collected 1h, 2h, 3h, 5h, 8h, 10h, 12h and 24h after the injection and the fluorescence signal was detected using an IVIS SPECTRUM *in vivo* imaging system (Xenogen, USA). Cy5 was excited by a 640 nm laser and collected between 650 and 680 nm while DiR excites at 745 nm and emits at 800 nm.

Cell culture and tumor model construction.

Five different cell lines were used in this study. 4T1, HUVEC, PC-3 and BT474 human breast cancer cells all have a high expression of both integrin αvβ3 and neuropilin-1 (NRP1) receptor. HeLa cells that express integrin αvβ3 but not NRP1 were used as the negative control.[4, 5] 4T1 cells were cultured in RPMI 1640 medium (Hyclone) supplemented with 10% fetal bovine serum

(Gibco). The HUVEC, PC-3 and HeLa cells were cultured in DMEM/High glucose (Hyclone) medium supplemented with 10% fetal bovine serum (Gibco). The BT474 cells were cultured in SFM4MAB medium supplemented with 10% fetal bovine serum (Gibco).

For the construction of 4T1 orthotopic tumors, 1×10^6 cells were orthotopically injected into the 5−7 week-old female BALB/c mice. For the construction of PC-3 and HeLa xenograft tumors, 1×10^7 cells were injected into the right/left flank of the 5−7 week-old female BALB/c nude mice. When the tumors were approximately 150 mm^3 in size, the *in vivo* fluorescence imaging experiments were conducted.

Western blot analysis.

The cells were collected and lysed with RIPA buffer (Solarbio, China) containing 1 mM phenylmethanesulfonyl fluoride (PMSF) (Solarbio, China). Protein samples (50-80 μg) were electrophoresed on 6% sodium dodecyl sulfate–polyacrylamide gels, and then transferred onto a polyvinylidene fluoride (PVDF) membrane. After pre-incubated in block solution at room temperature for 1 h, the PVDF membrane was incubated with rabbit anti-human NRP1 monoclonal antibody (1:1,000) for 2 h at room temperature. After being washed 3 times with Tris-buffered saline (TBS) containing 0.5% Tween-20 (Solarbio, China) for 10 min, the membrane was further incubated with goat anti-rabbit IgG secondary antibody (1:10,000) for 60 min at room temperature. Immunoreactive proteins were visualized using SuperSignal West Pico Chemiluminescent Substrate (Thermo Scientific, USA).

Confocal fluorescence imaging study.

For confocal fluorescence imaging, approximately 1×10^5 cells (in 1 mL culture medium) were seeded in confocal dishes and cultured overnight. The nuclei were stained with Hoechst 33342 at the concentration of 1 mM for 10 min. Then the cells were incubated with Propidium Iodide (PI, 0.5

mM) or PAD (5 μM DOX) and LipoCy5 nanoparticles (0.1 μM Cy5) at 37℃ for 2 h, respectively. The cells were washed three times with PBS prior to observation. The confocal fluorescence imaging was performed on an Olympus FV1000-IX81 confocal-laser scanning microscope. For DOX, a FV5-LAMAR 488 nm laser was used for excitation, and the emission was collected between 520 and 620 nm. Cy5 was excited by a 640 nm laser and collected between 650 and 680 nm. Hoechst 33342 was excited by a FV5-LD405-2405 nm laser and collected at the range of 422 to 472 nm. All parameters of the microscope were set to be the same for comparisons of different cells with different treatments.

Cytotoxicity analysis of LipoDOX coadministered with PADiR.

The cell viability assays were conducted by seeding PC-3 cells in 96-well plates at the density of 1×10^4 cells/well and culturing for 24 h before experiments. When the cells had reached 70% confluence, they were incubated with DOX, LipoDOX, iRGD+LipoDOX and PADiR+LipoDOX at different DOX concentrations for 20 h. The number of viable cells was determined using MTT assay. Briefly, the MTT solution was added to each well (100 μL/well) and incubated for 4 h. The solution was then carefully removed and 200 μL of dimethyl sulfoxide (DMSO) was added to each well. After 5 minutes of vibration mixing, the absorption at 570 nm was measured with an ELISA reader and the results were presented as mean ± S.D. % (n = 4) with the control group as 100% viability.

***In vitro* tumor penetration study.**

The in vitro tumor penetration efficacy of LipoDOX coadministered with PADiR peptosomes was studied using multicellular tumor spheroid (MCTS) model. Briefly, 200μL/well of cell suspension (1×10^4 cells/mL) was transferred into the ultra-low attachment (ULA) 96-well round bottom plate. Then the plate was transferred into a cell incubator (37 ºC, 5% CO_2) and incubated for

about 5 days to form the spheroids.[6] When the diameter of MCTS reached 500 μm, the culture medium was replaced with fresh medium supplemented with DOX, LipoDOX, PADiR+LipoDOX at the DOX concentration of 10 μg/mL. The MCTS were further incubated for 5 h to allow the penetration of the nanoparticles into the spheroids. The penetration and distribution of DOX in the spheroids were examined using confocal fluorescence imaging.

***In vivo* distribution analysis of nanoparticles and molecules coadministered with APPA self-assembled peptosomes.**

The fluorescent TRITC-containing APPA peptosomes (PAT) were prepared as the procedure mentioned above. All animal experiments were conducted in accordance with the institutional guidelines approved by the Institutional Ethical Committee of Animal Experimentation of National Center for Nanoscience and Technology. Animals received care in accordance with the Guidance Suggestions for the Care and Use of Laboratory Animals. The 5−7 week-old female BALB/c mice bearing 4T1 orthotopic tumors with the size of about 150 mm^3 were divided into 10 groups with 3 mice in each group for *in vivo* fluorescence imaging. The mice in group No. 1 to No. 4 were injected with PAT (100 μL), PCDiR (100 μL), PAT (100 μL)+PCDiR (100 μL) and iRGD (4 μmol/kg)+PCDiR (100 μL), while the mice in group No. 5 to No. 10 were injected with LipoCy5 (100 μL), PAD (DOX: 0.1 μmol)+LipoCy5 (100 μL), iRGD (4 μmol/kg)+LipoCy5 (100 μL), FreeCy5 (100 μL), PAD (DOX: 0.1 μmol)+FreeCy5 (100 μL) and iRGD (4 μmol/kg)+FreeCy5 (100 μL) *via* tail vein with the Cy5 concentration at 2 μM, respectively. Before the injection, the two compounds were mixed together for the coadministration (Figure S4). After the injection, the fluorescence images of the mice for detecting DiR probes were acquired (excitation: 745 nm, emission: 800 nm) using an IVIS SPECTRUM *in vivo* imaging system (Xenogen, USA) at different time points. For detection of Cy5, the excitation wavelength was set at 640 nm and collected at 680

nm. After 8 h of injection, the mice were sacrificed and the main organs were harvested for *ex vivo* imaging.

PAD peptosome coadministration with magnetosome and Fe_3O_4 for magnetic resonance imaging (MRI).

The 5−7 week-old female BALB/c nude mice bearing PC-3 xenograft tumors with the size of about 150 mm^3 were divided into 5 groups with 3 mice in each group for MRI imaging. The mice of each group were injected with magnotosome (Mag), PCD (DOX: 0.1 μmol)+Mag, iRGD (4 μmol/kg)+Mag, PAD (DOX: 0.1 μmol)+Mag and PAD+Fe3O4 with the amount of Mag or Fe3O4 at 25 mg/kg. The two compounds were mixed together before the coadministration. The T_2-weighted MRI imaging was conducted using a Bruker 7.0 T MR imaging system (Ettlingen, Germany) with the strength field of 7.0 T at 0 h, 2 h, 4 h, 5.5 h and 7 h after the coadministration. Seven hours after the coadminstration, the mice were sacrificed and the tumors were collected for Prussian blue staining analysis. To analyze the enrichment of small molecule, T_1 contrast agent Gd-DTPA (0.15 mmol/kg) alone or together with PAD peptosome were injected into three mice as the procedure described above, respectively. The T_1-weighted MR imaging were conducted at 20 min, 40 min and 2 h after the coadministration.

***In vivo* systemic permeability study.**

The 5−7 week old female BALB/c mice bearing 4T1 orthotopic tumors with the size of about 200 mm^3 were divided into three groups with three mice in each group. The mice in each group were intravenously injected with 100 μL of PBS containing 1 mg of Evans blue (EB), iRGD (4μmol/kg)+EB (1 mg) and PADiR (APPA:6 mg/kg)+Tra (1 mg), respectively. 1 h after the injection, the mice were sacrificed, the main organs and tumors were collected. For Evans blue

quantification, the dye was extracted from tissues in N,N-dimethylformamide for 24 h at 37℃ and the absorbance at 600 nm was quantified.

***In vivo* fluorescence signal colocalization of PADiR peptosome and the coadministered LipoCy5 liposome.**

The 5−7 week-old female BALB/c nude mice bearing HeLa xenograft tumors on their left flank and 4T1 xenograft tumors on the right flank with the tumor size of about 150 mm^3 were divided into two groups with three mice in each group for fluorescence imaging. The three mice in one group were injected with anti-NRP1 antibody (50 μg) to block the function of NRP1 and those in the other group were injected with IgG (50 μg) as the control via tail vein 15 min prior to the coadministration of PADiR and LipoCy5. Then the fluorescence signals of both Cy5 and DiR were acquired at 10 min, 2 h, 4 h, 6 h and 9 h with the position of the mice stay fixed.

Immunofluorescence.

The 5−7 week-old female BALB/c mice bearing 4T1 orthotopic tumors with the size of about 150 mm^3 were divided into four groups with three mice in each group. The mice in each group were intravenously administered with LipoCy5 (100 μL), PAT (100 μL), PAT (100 μL)+free Cy5 (2 μM, 100 μL) and PAT (100 μL)+LipoCy5 (Cy5: 2 μM, 100 μL), respectively. Six hours after the injection, the tumors were excised and slices were prepared as the procedure described in our previous study.[1] The cell membranes were stained with FITC labeled anti-Na+/K+ ATPase antibody and the nuclei with DAPI. The slices were observed with a confocal fluorescence microscope.

STED fluorescece imaging to visualize CoE process.

To investigate whether the coadministered nanoparticles were co-endocytosed into cells with APPA self-assembled peptosomes, stimulated emission depletion (STED) super-resolution fluorescence imaging microscopy was used to observe the cellular internalization process. To avoid

the fast fluorescence quenching during the experiment, DiD and DiI fluorescent probes were used to construct the fluorescent nanoprobes PADiD (DiD containing APPA self-assembled peptosomes) and PCDiI (DiI containing APPC self-assembled peptosomes). Then the nanoprobes were incubated with cells in confocal dishes for 0.5 h, 1 h and 2 h. After being washed 3 times, the cells were subject to STED imaging with DiI excites at 550 nm and emits at565 nm and DiD excites at 644 nm and emits at 665 nm.

4T1-H orthotopic tumor therapy using trastuzumab coadministration with peptosome.

To study the therapeutic efficacy of trastuzumab coadministered with APPA self-assembled peptosome on mice bearing 4T1-H orthotopic tumors, 4T1 cell line was transfected with pMH3-HER2 vector to construct the HER2 high expressing cell line (4T1-H). The expression of HER2 in the transfected cell line was analyzed by immunofluorescence staining as the procedure mentioned above. Then the 4T1-H cells were used to create the orthotopic tumor mouse model for the therapeutic study. The 5−7 week-old female BALB/c mice bearing 4T1-H orthotopic tumors with the size of about 60 mm^3 were divided into four groups with five mice in each. The mice in each group were intravenously injected with PBS, trastuzumab (Tra: 6 mg/kg), iRGD (4μmol/kg)+Tra (6 mg/kg), PADiR (APPA:6 mg/kg)+Tra (6 mg/kg) every other day, respectively. Fluorescence images were taken to show the distribution of Tra throughout the bodies using an IVIS SPECTRUM *in vivo* imaging system (Xenogen, USA). The body weights of each mouse were measured before and after each injection. The tumor volumes were measured with a digital caliper and estimated by the formula $(L\times W^2)/2$, where L is the longest and W is the shortest diameter of the tumor. For further studies and humane reasons, the mice were sacrificed when the tumor volume in PBS group reached 800 mm^3 after 6 injections. The livers, lungs, and kidneys were harvested for hematoxylin-eosin (H&E) staining. The distribution of Tra (Cy5 labeled) in the tumor tissue was

analyzed by immunofluorescence imaging. The H&E staining slices were observed with a light microscope (EVOS, Life Technologies).

PC-3 xenograft tumor treatment using LipoDOX coadministered with peptosome.

The 5−7 week-old female BALB/c nude mice bearing PC-3 xenograft tumors with the size of about 100 mm^3 were divided into four groups with five mice in each group. The mice in each group were intravenously injected with PBS, LipoDOX, iRGD (4 μmol/kg)+LipoDOX, PADiR (APPA:6 mg/kg)+LipoDOX with the DOX dosage of 5 mg/kg every 3 days, respectively. The tumor volumes were measured and calculated as the procedure mentioned above. For further studies and humane reasons, the mice were sacrificed when the tumor volume in PBS group reached 700 mm^3 after 5 coadministrations. The hearts, livers, lungs and kidneys were harvested and subjected to hematoxylin-eosin (H&E) staining. Apoptosis of tumor cells was evaluated by H&E staining and terminal deoxynucleotidyl transferase dUTP nick end labeling (TUNEL). The TUNEL-positive cells were quantified by observing five random sections of tumor slices from each group by confocal microscopy. The data were presented as mean ± S.D. (n=5).

Results

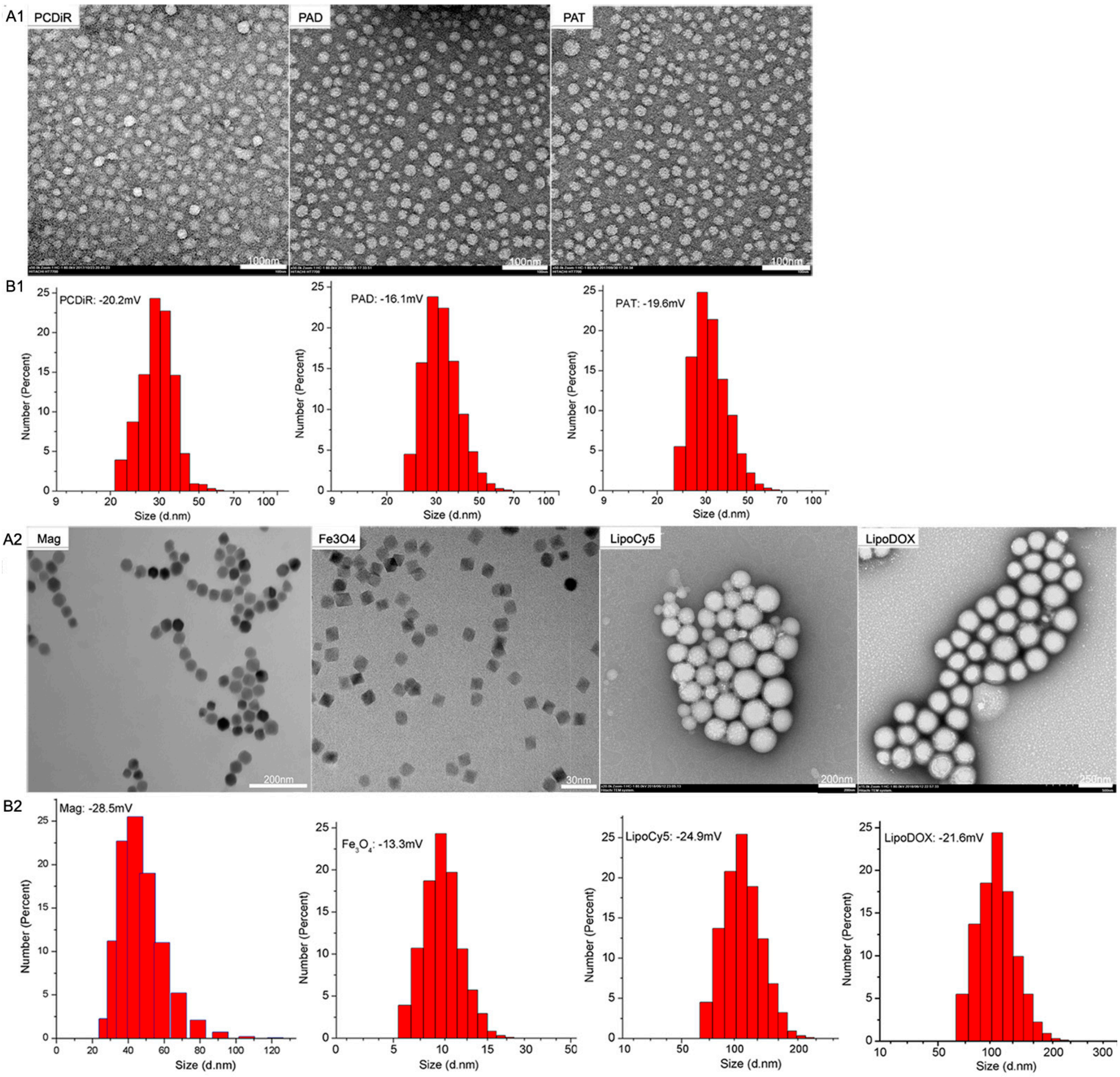

Figure S1. Characterization of PCDiR (DiR-containing APPC self-assembled peptosome), PAD (DOX-containing APPA self-assembled peptosome), PAT (TRITC-containing APPA self-assembled peptosome), magnetosome, Fe_3O_4, LipoCy5 (Cy5-containing liposome) and LipoDOX (DOX-containing liposome) used in this study. (A1, A2) TEM images of PCDiR, PAD, PAT, Magnetosome, Fe3O4, LipoCy5 and LipoDOX. (B1, B2) The size distribution and surface zeta potential of PCDiR, PAD, PAT, Magnetosome, Fe_3O_4, LipoCy5 and LipoDOX nanoparticles.

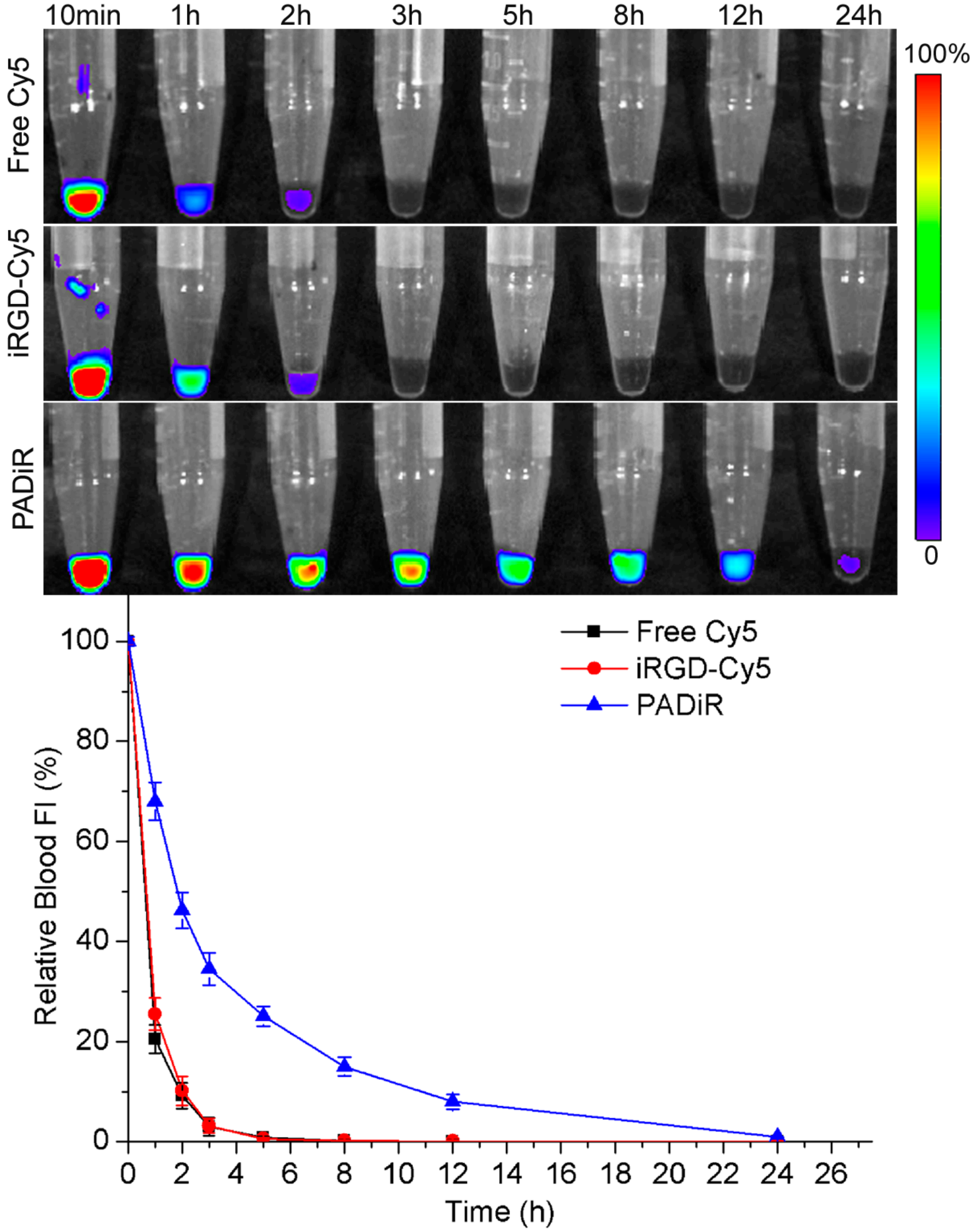

Figure S2. The blood clearance rate comparison of iRGD and APPA self-assembled peptosome. (A) The fluorescence images of the blood of BALB/c nude mice that were intravenously injected with free Cy5, iRGD-Cy5 and PADiR, respectively. The blood was collected and imaged at 10 min, 1 h, 2 h, 3 h, 5 h, 8 h, 12 h and 24 h after injection. (B) Quantification of blood fluorescence intensity (FI) over time relatively to the intensity observed at 10 min.

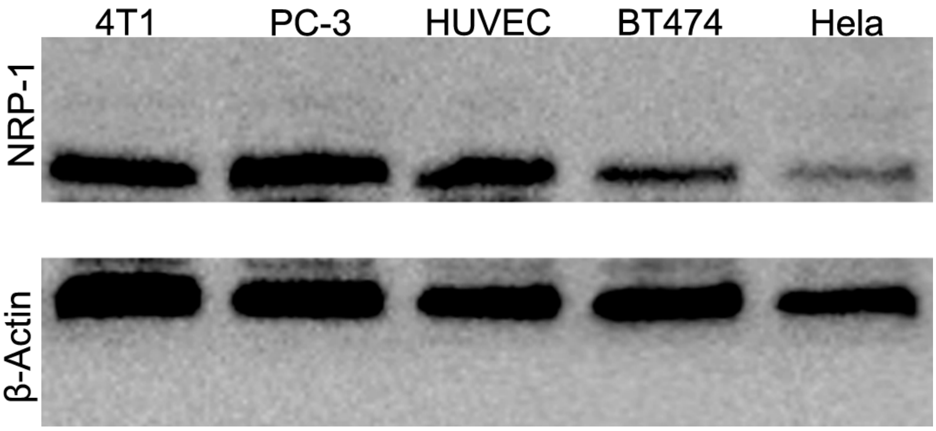

Figure S3. Western blot analysis of NRP1 receptors in 4T1, PC-3, HUVEC, BT474 and HeLa cell lines used in this study.

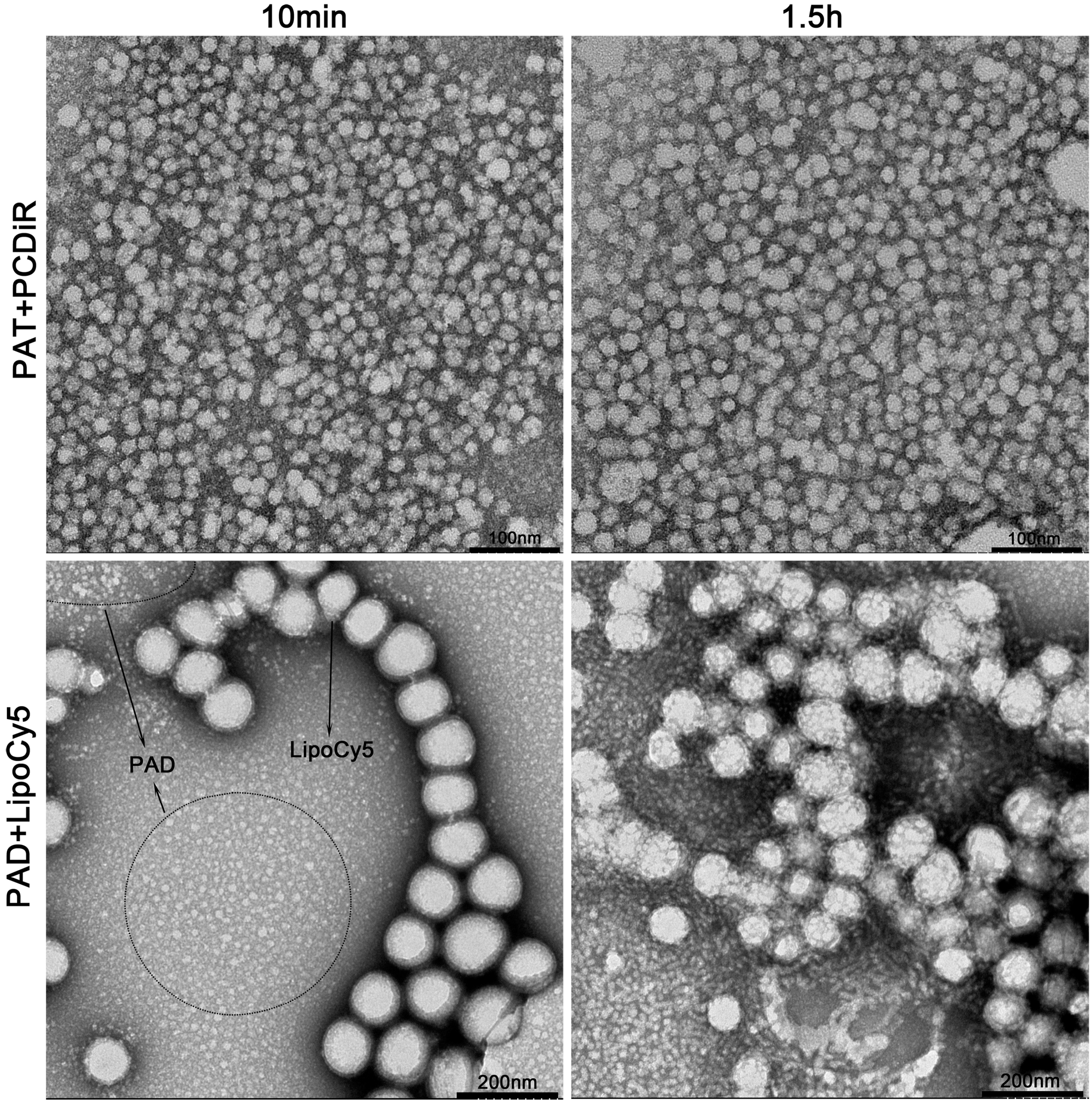

Figure S4. TEM images showing the morphology of PAT+PCDiR and PAD+LipoCy5 at 10 min and 1.5 h after mixing of the two compounds.

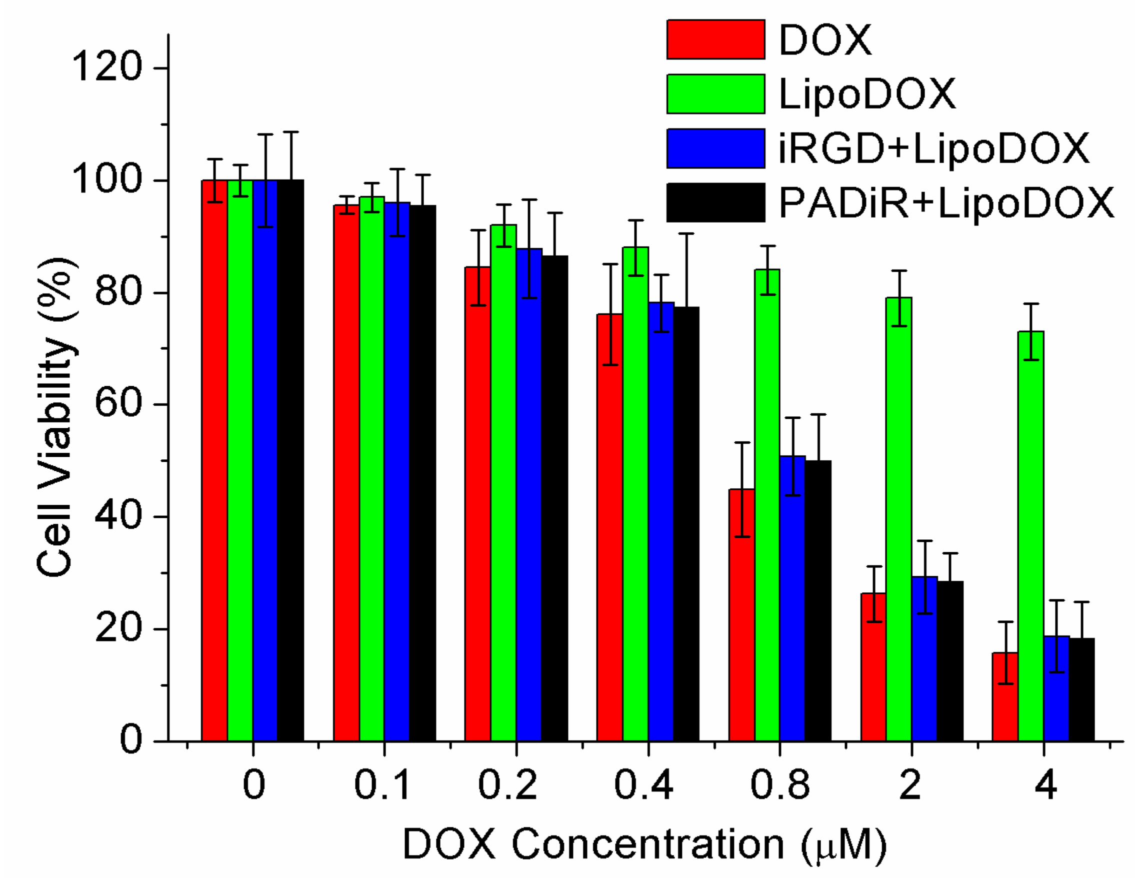

Figure S5. Cytotoxicity of LipoDOX coadministered with peptosome PADiR to PC-3 cells compared with using DOX and LipoDOX alone as well as LipoDOX coadministered with iRGD using the MTT assay.

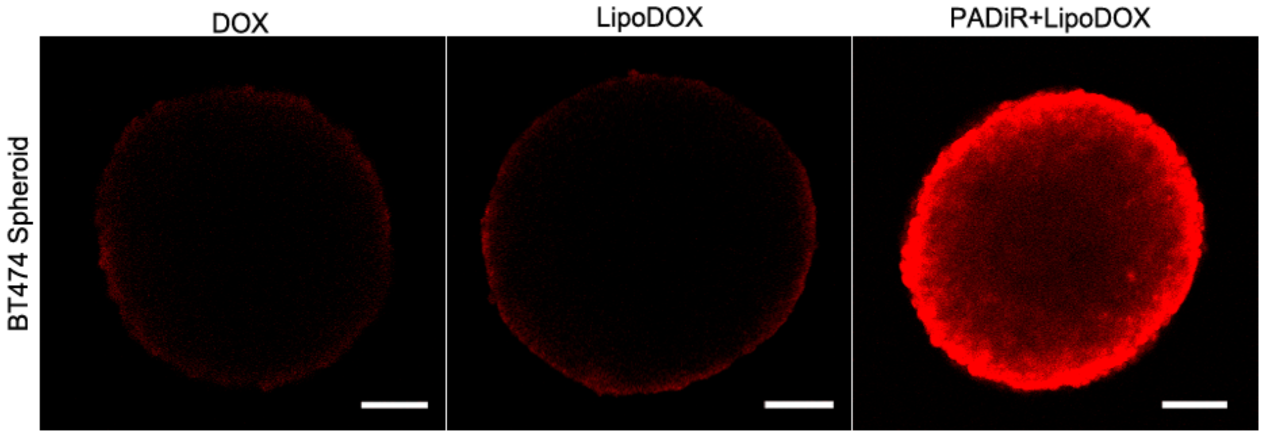

Figure S6. Confocal fluorescence images of BT474 multicellular tumor spheroids (MCTS) incubated with DOX, LipoDOX and PADiR+LipoDOX. Scale bars: 100 μm (excitation: 488 nm and emission: 594 nm).

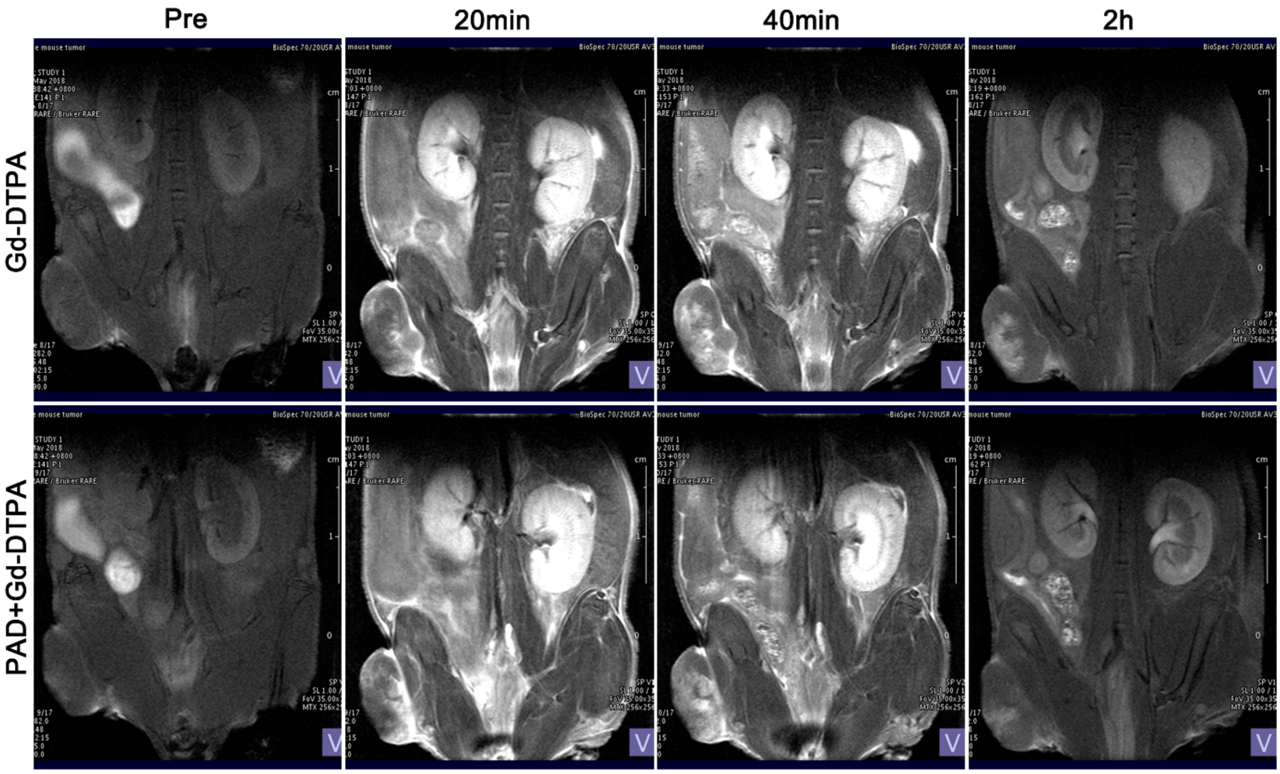

Figure S7. T_1-weighted MRI imaging (7.0 T) of BALB/c nude mice bearing PC-3 xenograft tumors before and 20 min, 40 min and 2 h after injection of Gd-DTPA (0.15 mmol/kg) alone, or coadministration of Gd-DTPA (0.15 mmol/kg) with PAD peptosome.

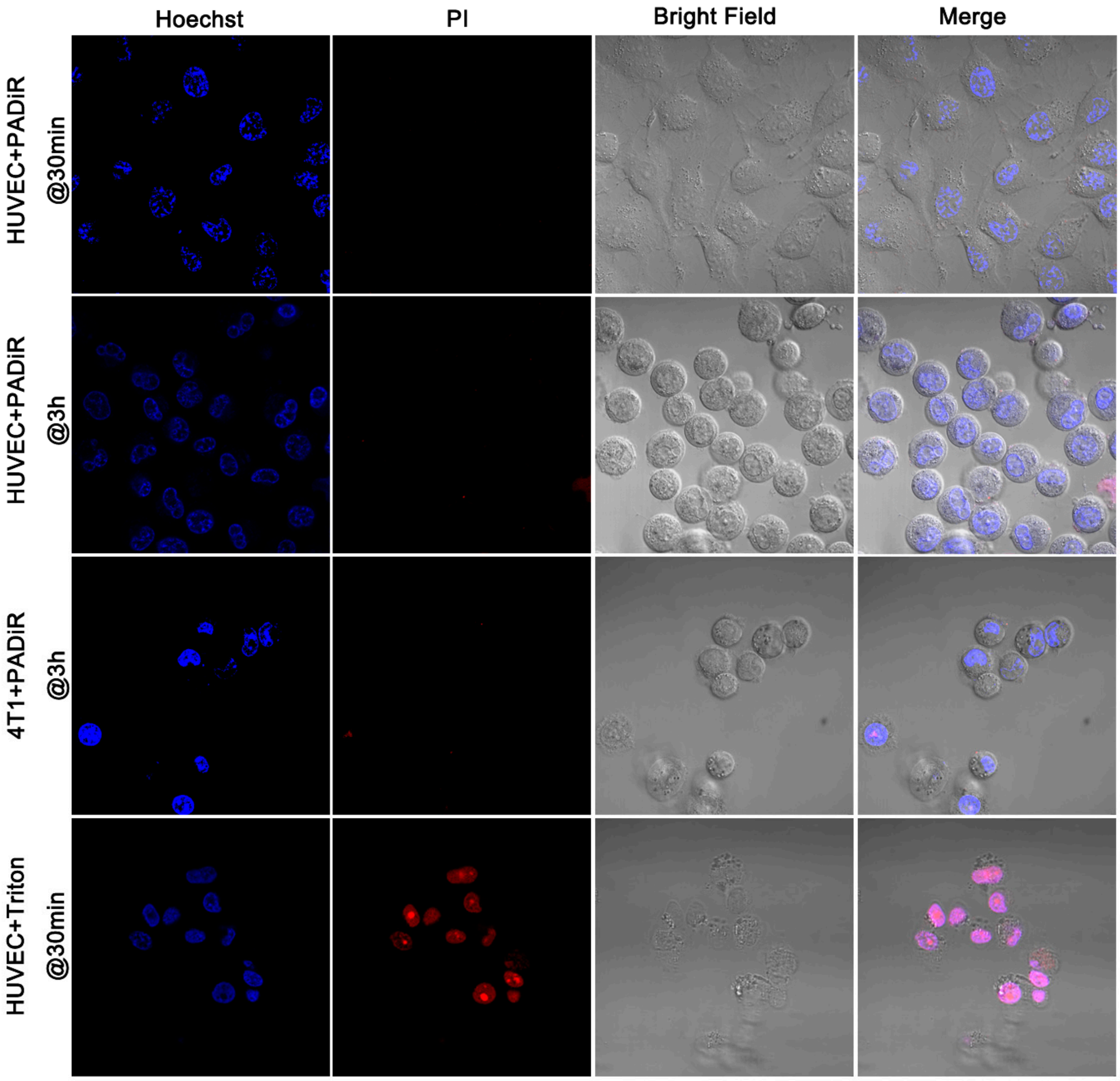

Figure S8. Cell membrane integrity of HUVEC and 4T1 cells using PI staining. The nucleus were stained with Hoechst (blue), then the cells were further incubated with PADiR peptosome and PI (red) at 37 ºC for different time duration. As the positive control, the HUVEC cells were incubated with 0.3% TritonX-100 for 10 min before the incubation with Hoechst and PI. The fluorescence images were taken after 30 min and 3 h of incubation.

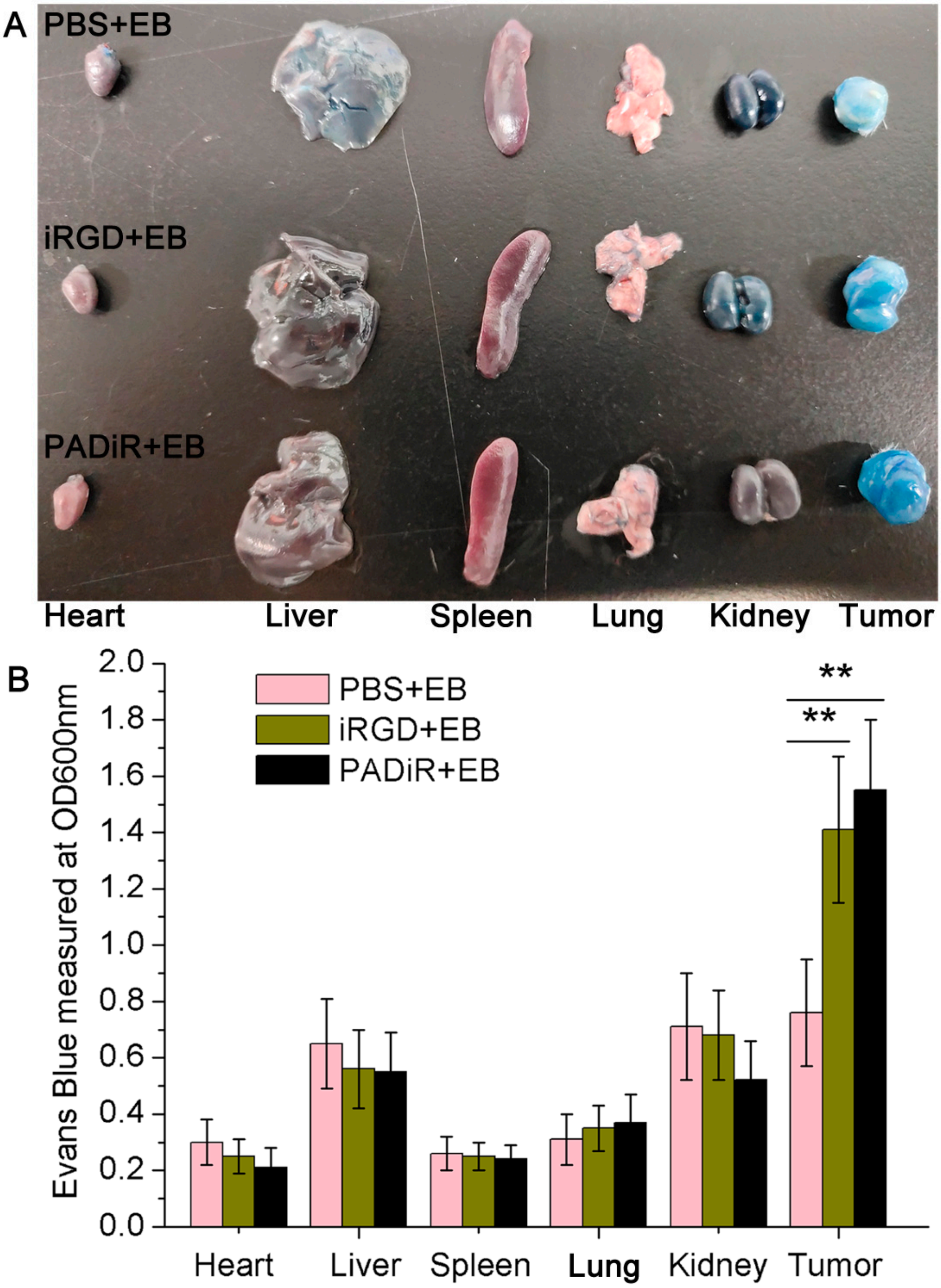

Figure S9. Tumor-specific enrichment of Evans blue in 4T1 orthotopic tumor model. (A) Macroscopic appearances of tumors and main organs from the mice coadministered with PBS, iRGD and PADiR, respectively. (B) Quantification of Evans blue in the tissues shown in (A). Results are shown as mean absorbance ±SD ($n = 3$), $^{**}P < 0.01$.

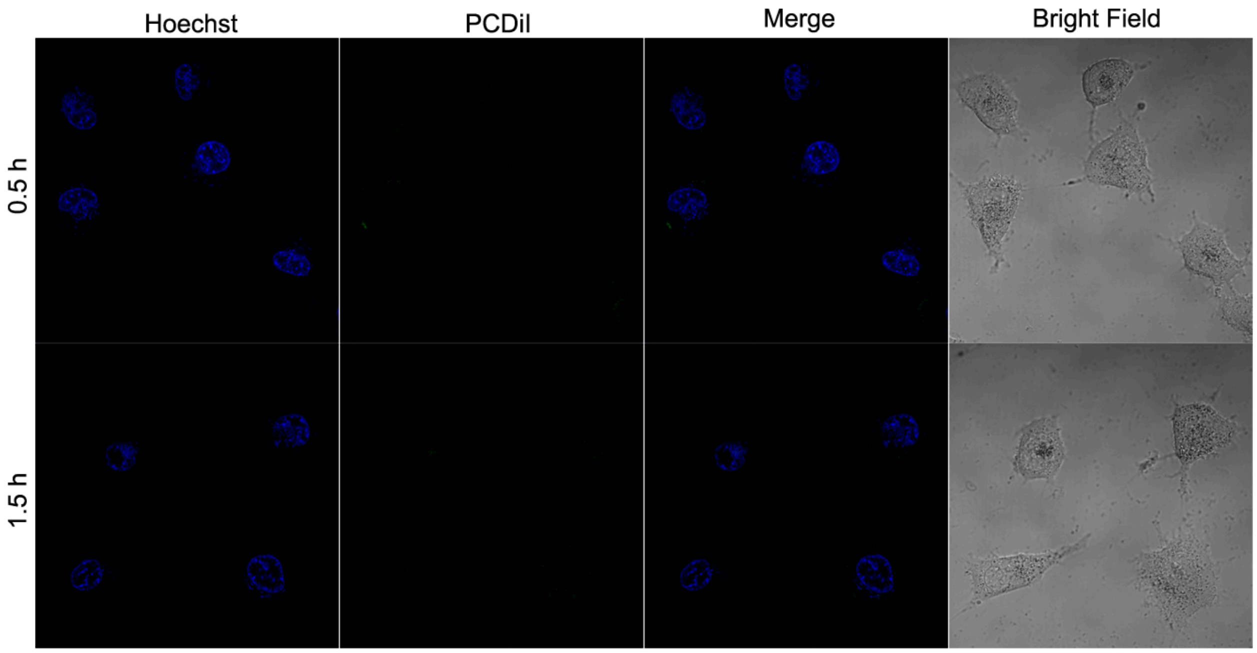

Figure S10. Fluorescence images of PC-3 cells showing no binding of PCDiI (DiI-containing APPC peptosomes) to the cells. Cells were incubated with PCDiI at 37 ºC for 0.5 h and 1.5 h before the fluorescence images were taken. (DiI excites at 550 nm and emits at565 nm)

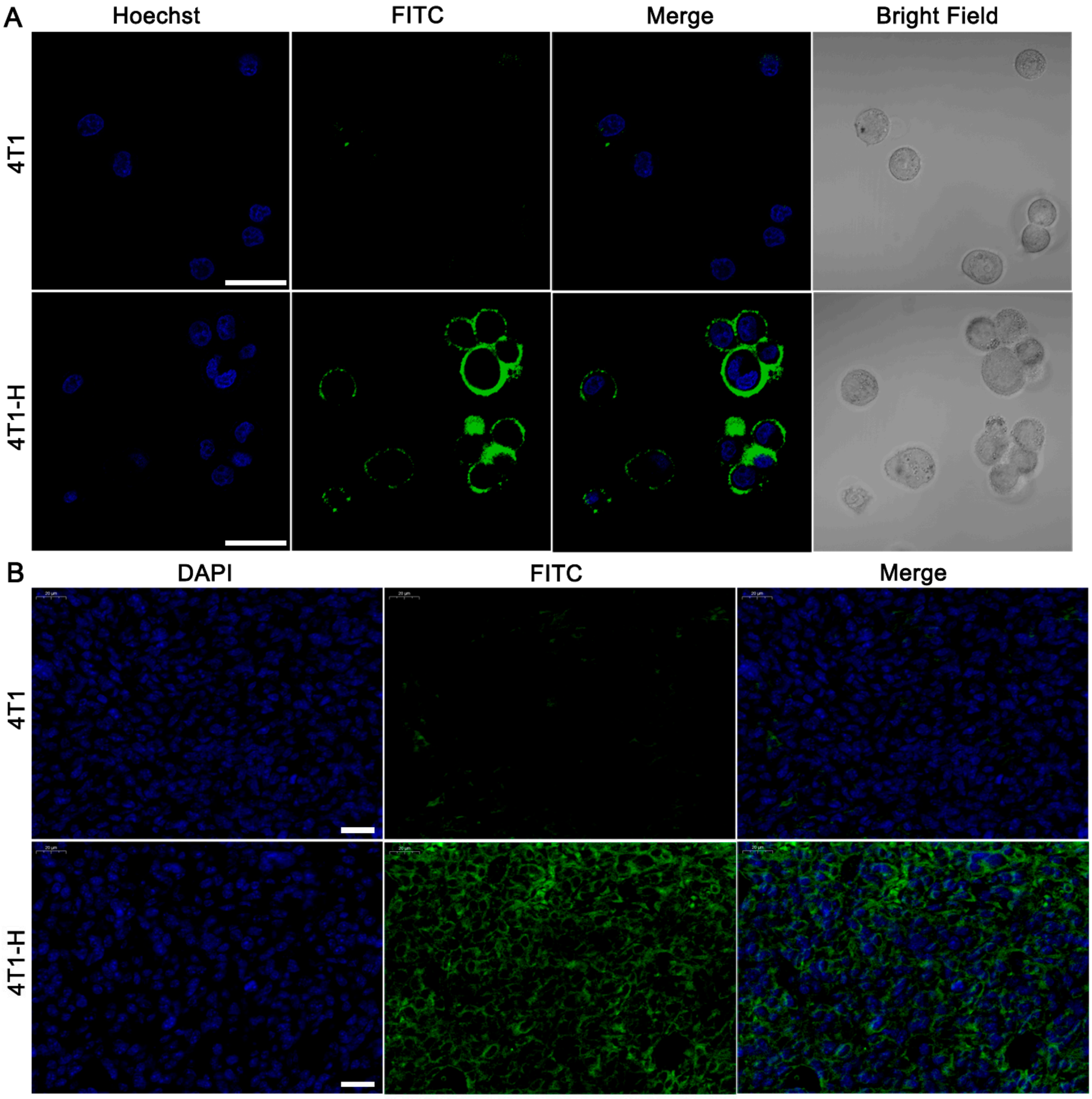

Figure S11. Expression of HER2 in 4T1 and 4T1-H cells and their corresponding orthotopic tumors. (A) Confocal fluorescence images of 4T1 and 4T1-H after incubating with anti-HER2 antibody (FITC labeled, green) for 20 min. (B) Immunofluorescence staining of 4T1 and 4T1-H orthotopic tumor slices using anti-HER2 antibody (FITC labeled, green). Nuclei are shown in blue. Scale bars: 20 μm.

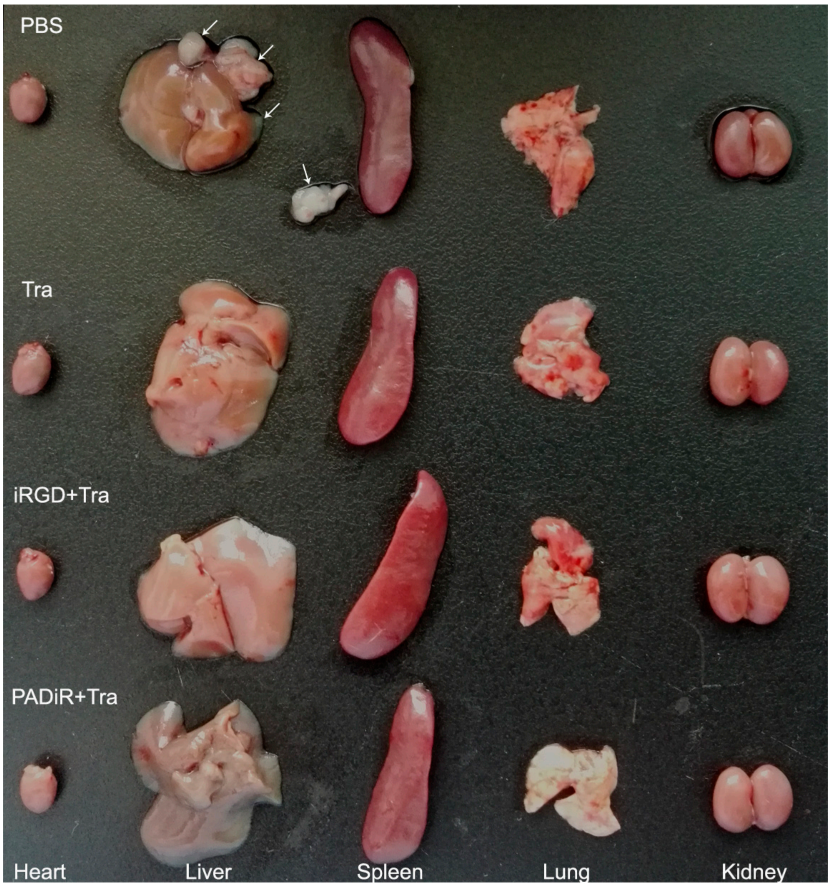

Figure S12. Images of main organs from the mice bearing orthotopical 4T1-H tumors 21 days after treatment with PBS, Tra(trastuzumab), Tra coadministered with iRGD and Tra coadministered with PADiR peptosomes. The white arrows indicate the metastatic tumors.

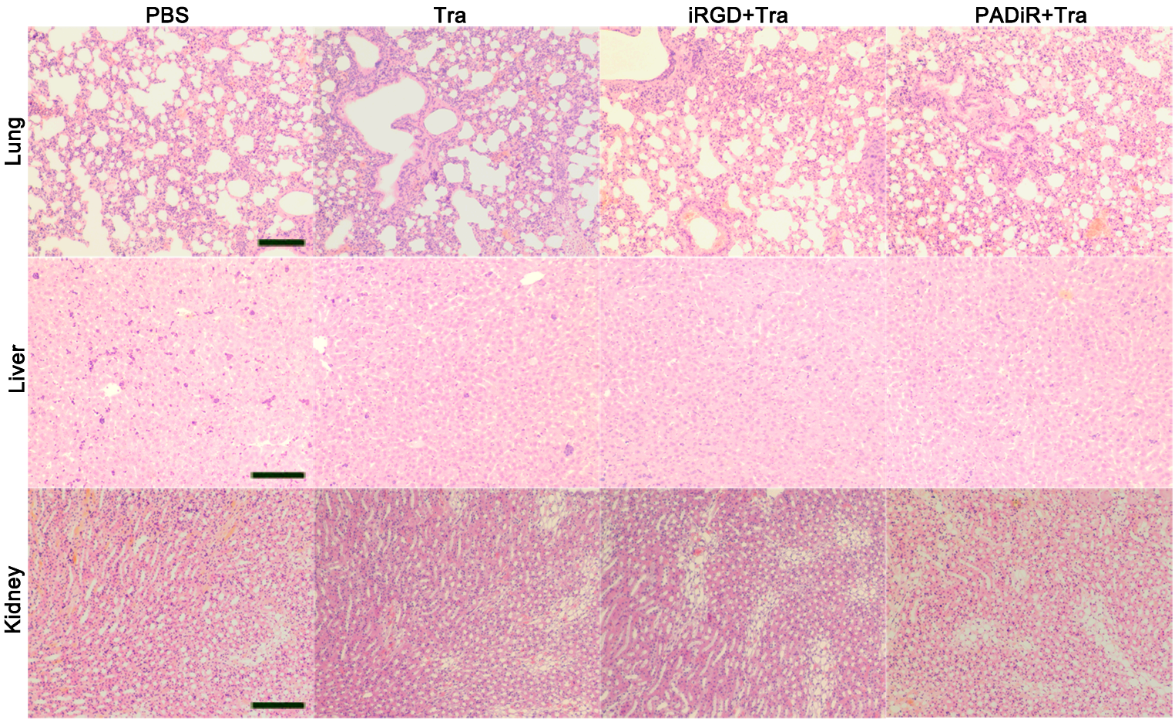

Figure S13. H&E staining of the lung, liver and kidney tissue slices from the mice bearing orthotopical 4T1-H tumors 21 days after treatment with PBS, Tra (trastuzumab), Tra coadministered with iRGD and Tra coadministered with PADiR peptosomes. Scale bars: 100 µm.

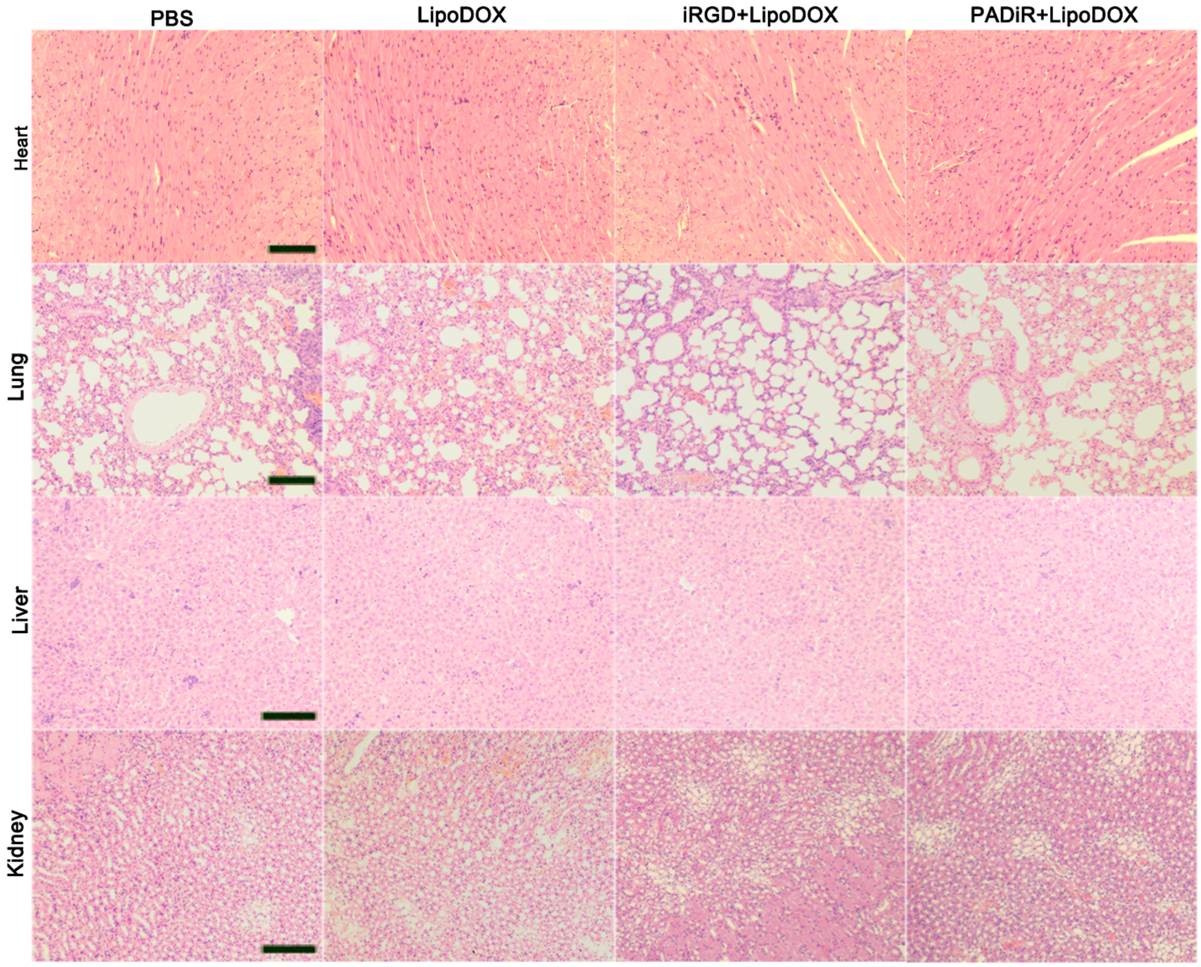

Figure S14. H&E staining of the heart, lung, liver and kidney tissue slices from the mice bearing PC-3 xenograft tumors 29 days after the treatment with PBS, LipoDOX, LipoDOX coadministered with iRGD and Tra coadministered with PADiR peptosomes. Scale bars: 100 μm.

References

1. Xiang, Z. Yang, X.; Jiang, G.; Fan, D.; Geng, L.; Wang, H.; Hu, Z.; Fang, Q., Design of a Simple and Practical Nanosystem Coordinates Tumor Targeting and Penetration for Improved Theranostics. *Advanced Theraputics* 2018, 1800107(1-9).

2. Xiang, Z.; Yang, X.; Xu, J.; Lai, W.; Wang, Z.; Hu, Z.; Tian, J.; Geng, L.; Fang, Q., Tumor detection using magnetosome nanoparticles functionalized with a newly screened EGFR/HER2 targeting peptide. *Biomaterials* 2017, 115, 53-64.

3. Fang, R. H.; Aryal, S.; Hu, C. M.; Zhang, L., Quick synthesis of lipid-polymer hybrid nanoparticles with low polydispersity using a single-step sonication method. *Langmuir : the ACS journal of surfaces and colloids* 2010, 26, 16958-62.

4. Pang, H. B.; Braun, G. B.; Friman, T.; Aza-Blanc, P.; Ruidiaz, M. E.; Sugahara, K. N.; Teesalu, T.; Ruoslahti, E., An

endocytosis pathway initiated through neuropilin-1 and regulated by nutrient availability. *Nature communications* 2014, 5, 4904.

5. Wang, T.; Wang, D.; Liu, J.; Feng, B.; Zhou, F.; Zhang, H.; Zhou, L.; Yin, Q.; Zhang, Z.; Cao, Z.; Yu, H., Acidity-Triggered Ligand-Presenting Nanoparticles To Overcome Sequential Drug Delivery Barriers to Tumors. *Nano letters* 2017, 17, 5429-5436.

6. Vinci, M.; Box, C.; Eccles, S. A., Three-dimensional (3D) tumor spheroid invasion assay. *Journal of visualized experiments : JoVE* 2015, e52686.
